# A theoretical and experimental investigation on a volume coil with slotted end-rings for rat MRI at 7 T

**DOI:** 10.1007/s10334-023-01096-w

**Published:** 2023-05-15

**Authors:** Sergio Solis-Najera, Rodrigo Ruiz, Rodrigo Martin, Fabian Vazquez, Oscar Marrufo, Alfredo Odon Rodriguez

**Affiliations:** 1 Departamento de Fisica, Facultad de Ciencias, UNAM, 04510 Mexico City, Mexico; 2 Department of Electrical Engineering, UAM Iztapalapa, 09340 Mexico City, Mexico

**Keywords:** Magnetic resonance imaging, RF coils, Electromagnetic fields, High field, Preclinical

## Abstract

**Objective:**

A volume coil with squared slots-end ring was developed to attain improved sensitivity for imaging of rat’s brain at 7 T.

**Material and Methods:**

The principles of the high cavity resonator for the low-pass case and the law of Biot-Savart were used to derive a theoretical expression of $B_1/i$. The slotted-end ring resonator showed a theoretical 2.22-fold sensitivity improvement over the standard birdcage coil with similar dimensions. Numerical studies were carried out for the electromagnetic fields and specific absorption rates for our coil and a birdcage coil loaded with a saline-filled spherical phantom and a digital brain of a rat.

**Results:**

An improvement of the signal-to-noise ratio (SNR) can be observed for the slotted volume coil over the birdcage regardless of the load used in the electromagnetic simulations. The specific absorption rate simulations show a decrement for the digital brain and quite similar values with the saline solution phantom. Phantom and rat’s brain images were acquired at 7 T to prove the viability of the coil design. The experimental noise figure of our coil design was four times less than the standard birdcage with similar dimensions, which showed a 44.5% increase in experimental SNR.

**Discussion:**

There is remarkable agreement among the theoretical, numerical and experimental sensitivity values, which all demonstrate that the coil performance for MR imaging of small rodents can be improved using slotted end-rings.

## Introduction

In vivo imaging of mice and rats is soundly established as a component of preclinical and translational biomedical research [1–5]. The biomedical research community has recognized the unique power of magnetic resonance imaging (MRI) for in vivo measures in small animals [6]. Advances in RF technology have provided a boost to progress of research-dedicated MRI systems equipped with strong magnets (> 7 T) for small animal imaging. The RF receive coil is vital in determining high signal-to-noise ratio (SNR), and image quality increases with SNR, hence coil selection is critical for rodent MRI experiments. Volume coils for small animal investigations with MRI are a particularly popular choice for a number of reasons [7, 8]. In particular, birdcage coils have been a popular design for a number of years. This type of RF coil offers a convenient geometry because it can generate an excellent field uniformity, sensitivity, and natural ability to operate in quadrature. Additionally, they may be placed coaxially with the bore of the magnet for easy loading and unloading of rodents. The birdcage coil is still an important subject of study as shown by recent results [8–15]. The design of dedicated RF coils is key to achieving the best preclinical and experimental results for MRI. The end rings of the birdcage coil are an important design aspect as they can modify the intensity and homogeneity [7, 10, 13–15]. The principles of the cavity resonator proposed by Mansfield et al. [16] offer an approach to improve the intensity and homogeneity of volume coils, with end rings composed of uniformly distributed slots forming a symmetrical distribution.

In this paper, we developed a coil design based on the RF coil reported in [17] and used for whole-body imaging of rats at 7 T. The coil designed proposed here is composed of squared slots end rings and reduced number of rungs to decrease specific absorption rate (SAR) [18]. We derived an expression of the sensitivity based on the low-pass cavity resonator and the law of Biot-Savart to investigate the coil performance, and experimentally validate this theoretical frame. This coil design is intended for MR imaging of a rodent’s brain. The present work is based on a preliminary previous report [19] to experimentally improve the coil sensitivity using this slotted-end ring layout.

**Fig. 1:**
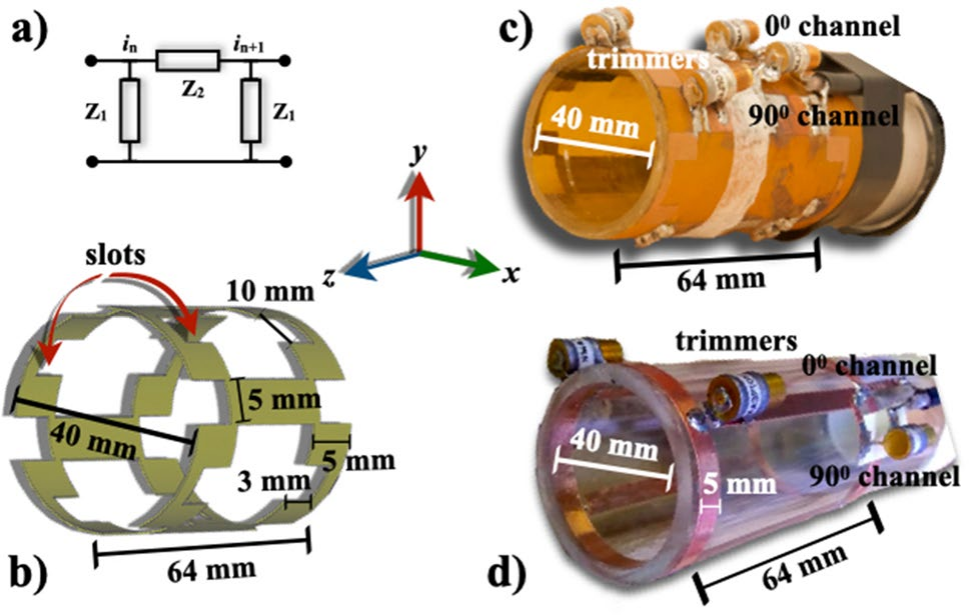
**a** A $\pi$-section of a lumped parameter transmission line, **b** schematic view of the slotted-end ring volume coil, photographs of constructed squared-slot end ring coil showing dimensions and passive electronic components **c**, and birdcage coil used for comparison purposes **d**

## Method

### End-rings comparison

There is an important difference between the end-rings in a standard birdcage coil and the modified design of the cavity resonator presented here. Figure 1 shows a schematic and photograph of the coil proposed in this research. To investigate the effect of the squared-slot end rings, we used the formalism for the physical principles of the cavity resonator developed by Mansfield et al. for the low-pass topology [16]. From this and following the calculation of the total current expected to flow in each half sheet [20], we have:

1 $$i_{0}^{2} = i_{{{\text{slot}}}}^{2} \frac{R}{{r\sum\nolimits_{{n = 1}}^{N} {\cos ^{2} } \left( {n\Theta } \right)}}$$

where $i _0$ is the maximum current, $i _\textrm{slot}$ is the drive current, *R* is the input resistance and *r* is the slot resistance, and $\sum\limits_{{n = 1}}^{N} {\cos ^{2} } \left( {n\Theta } \right) = \frac{N}{2}$, and

2 $$\begin{aligned} R=\frac{2}{Nr}Z_{0}^{2} \end{aligned}$$

where $Z_{0}$ is the characteristic impedance which is a function of the slot and rung impedance, and *N* is the number of rungs. To compute *r*, we use *Q*, the quality factor of the low-pass cavity resonator:

3 $$\begin{aligned} Q=\frac{2\pi }{Nr}Z_{0} \end{aligned}$$

Substituting for the resistances *R*, *r* using Eqs. (2) and (3) in Eq. (1), we obtain,

4 $$\begin{aligned} \displaystyle i_{\textrm{0}}=i_{\textrm{slot}}\frac{NQ}{\pi } \end{aligned}$$

the ratio $\frac{i_0}{i_\mathrm {_{slot}}}$ in Eq. (4) depends on the number of rungs and the quality factor. For comparison purposes, we proceeded similarly as above, so the birdcage end rings can be studied using the intensity of the rung currents [20]:

5 $$\begin{aligned} \displaystyle \textrm{i}_{\textrm{0}}={i}_{\textrm{bc}}\,{2\sin \left( \frac{\pi }{N}\right) } \end{aligned}$$

where bc refers to the birdcage coil. Then, assuming that $i_{\textrm{bc}}=i_\mathrm {_{slot}}$ and $i_{\textrm{0}}= \textrm{i}_\textrm{0}$, and combining eq. (4) and Eq. (5):

6 $$\begin{aligned} \displaystyle Q={2\pi }\sin \left( \frac{\pi }{N}\right) \end{aligned}$$

In particular, for the 4-rung coil layout,

7 $$\begin{aligned} \displaystyle i_{\textrm{0}}=1.4\,i_{\textrm{slot}} \end{aligned}$$

Once we have computed the currents for the birdcage coil and the cavity resonator, we can now compare the transverse magnetic field $\textrm{B}_{1}$. The $\textrm{B}_{1}$ at the coil’s isocenter relative to the end-rings current for our coil design with 4 rung is [20, 21]:

8 $$\begin{aligned} \displaystyle \textrm{B}_{\mathrm {\textrm{1}slot}}=2.2\frac{i_{\textrm{slot}}\left( l^{2}+2d^{2}\right) }{d\left( l^{2}+d^{2}\right) ^{3/2}} \end{aligned}$$

where *l* and *d* represent the length and the diameter of the volume coil, respectively, and Eq. (8) was computed with no shielding. A more detailed derivation of the sensitivity expression for a birdcage coil can be found in Refs. [22, 23].

### Electromagnetic field simulations

**Fig. 2:**
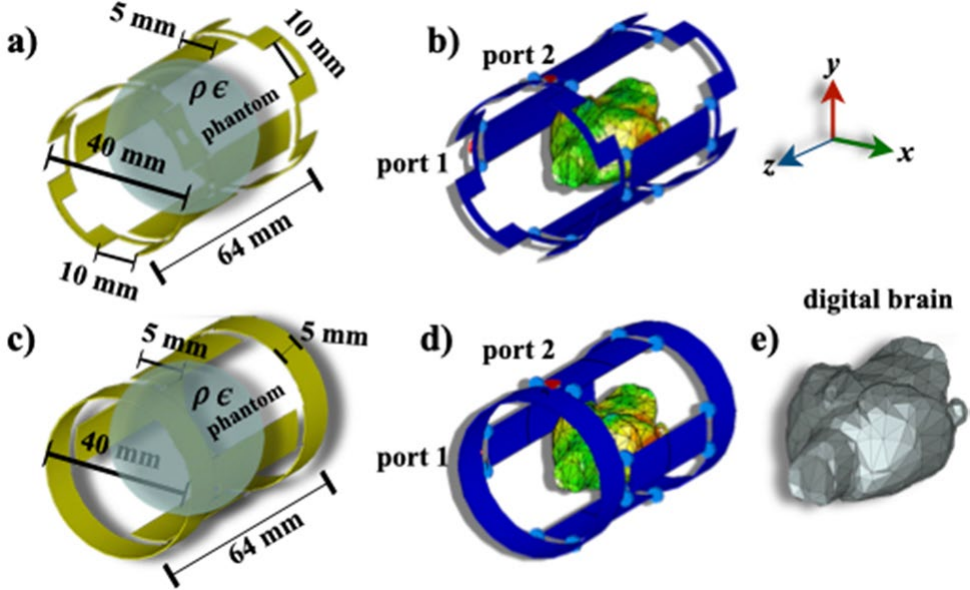
The simulation setups for both coils and their dimensions and the cylinder phantom are shown in **a** and **c** while the rat’s digital phantom is shown in **b** and **d**. The digital phantom used in the electromagnetic and SAR simulations is shown in **e**

The electromagnetic field simulations of RF coils can serve to guide the development of specific designs for specific applications and demonstrate how this coil design interacts with the sample to be imaged [24]. The commercial software CST Microwave Studio (CST MICROWAVE STUDIO, CST GmbH, Darmstadt, Germany) was used to calculate the electromagnetic fields. We have experimentally validated this commercial code with a birdcage coil for whole-body MRI of rats at 7 Tesla [18]. To numerically calculate the electromagnetic fields of the slotted-end rings coil (Fig. 1b, perfect electric conductors (PEC) were assumed, and together with a four-leg configuration and a saline-solution spherical phantom were used. The phantom properties were $\sigma _{\text {solution}}=0.55$ S/m, $\varepsilon =78.4$, $\rho =$ 998 kg/m$^{3}$, and $\upmu$ = 0.999991. A 1 V sinusoidal feed was applied, and the source and conductor impedances were set to 50 $\Omega$ (pure resistive). To calculate more realistic results, the rat’s brain phantom ($\sigma _{\text {brain}}=0.527133$ S/m, $\varepsilon =70$, $\rho =$ 1030 kg/m^3^, and $\upmu$ = 1) reported in [18, 24] was also used. This rat’s brain model is considered a voxel-based models constructed using digital volume arrays and boundary representation (BREP) models. These type of models offer an easy implementation and fast calculation within most commercial simulation codes. [25]. A safety evaluation of RF coils is especially important to protect the sample from heat and temperature increase [26]. This is mainly done by using RF simulations in a heterogeneous body to compute realistic spatial distributions of SAR. All SAR predictions were computed assuming 1 g averaging, the IEEE STD C$-$95.3.1-2010 method, 1 W input power and were performed with open boundary conditions defined in all directions. Figure 2a and c show the schematic used in the corresponding numerical assessments.

The calculations were terminated after simulations reached 4.23 pulse widths corresponding to a system energy decay of around – 30 dB for all cases, and the duration of the excitation was around 7.11 ms. Similarly, simulations were run for a birdcage with similar dimensions and the same configurations. The coils were excited in quadrature mode. The simulation setups, phantoms, and volume coils are shown in Fig. 2b and d.

### Coil prototype

To optimize SNR performance, the coil dimensions should match the size of a mouse while matching the homogeneous RF region. The size of the volume coil was chosen to accommodate rats while also taking into account that a 12 cm bore was available. For this study, our coil design was 64 mm in length, 40 mm in diameter, contained four rungs, and both end rings were composed of four equally spaced rectangular slots. Figure 1b and c show a schematic and a photograph of the coil. We designed our coil with four rungs to attenuate the SAR as experimentally shown by Martin et al. [18]. The coil dimensions give: $\mathrm {diameter/length}=0.625$ to avoid field homogeneity problems and to drastically affect the SNR. This result is in good concordance with Ref. [12, 22]. Our coil design is a low-pass resonator because $\frac{\lambda }{20}=50$ cm, where $\lambda$ is the wavelength at 300 MHz. The RF coil prototype was built on a semi flexible printed circuit board (Pyralux$^\circledR$circuit flexible material: thickness = 100 $\mu$m, $\epsilon =2.5$, $\tan (\delta )=0.002$. Dupont™, Inc. Wilmington, DE, USA) according to the specific coil configuration. Wapler et al. have conducted considerable investigations on a number of materials suitable to build MRI coils such as Pyralux$^\circledR$material [27]. This material has also been used to print coil arrays for clinical MRI [28]. This printed circuit board was mounted on an acrylic cylinder to form a volume coil. The prototype was tuned to 299.47 MHz (the proton frequency at 7 T) using nonmagnetic chip capacitors and trimmers. One 50 $\Omega$-coax cable was attached to each channel ($0^{0}$ and $90^{0}$ channels) for quadrature drive, tuning and matching. Rough tuning was achieved with eleven and six fixed-value chip capacitors (American Technical Ceramics, series ATC 100 B nonmagnetic) of 3.7 pF and 3.9 pF, respectively. 50-$\Omega$ matching and fine tuning was achieved using four nonmagnetic trimmers (Voltronics, Corp: 1–33 pF, NMAJ30 0736) two for each channel. The resonant frequency for each channel was measured using a network analyzer (Model 4396A, Hewlett Packard, Agilent Technologies, CA) as the return loss ($\textrm{S}_{11}$). After fine tuning and matching, both channels were decoupled at the desired frequencies by altering the balancing capacitor values. The quality factor (*Q*) of each channel in the coil was also experimentally determined by measuring the resonant frequency divided by the 3 dB bandwidth, $\Delta \omega$, with a quarter-wavelength coaxial cable at the input of the coil. The loaded *Q* value was measured while the coil was loaded with a saline-filled spherical phantom (3 cm diameter).

### Imaging experiments

To test the validity of this coil, spherical phantom images were acquired using a standard spin echo sequence. The acquisition parameters were: TE/TR = 25 ms/900 ms, FOV = 40 mm × 40 mm, matrix size = 256 × 256, slice thickness= 2 mm, NEX = 1. Additionally, images of mouse’s head were acquired using gradient echo sequence and the following acquisition parameters: TE/TR = 6 ms/400 ms, flip angle = $90^{0}$, FOV = 35 mm × 35 mm, matrix size = 256 × 256, slice thickness = 1 mm, NEX = 1. All MRI experiments were performed on a 7T/21 cm Varian imager equipped with DirectDriveTM technology (Varian, Inc, Palo Alto, CA) and, a SGRAD 205/120/HD gradient system capable of producing pulse gradients of 400 mT/m in each of the three orthogonal axes and interfaced to a VnmrJ 2.1B console. All procedures involving mice were approved by Bioethical Committee of the Division of Biological and Health Sciences at the Universidad Autonoma Metropolitana Iztapalapa.

### Noise factor

The RF penetration decreases when the coil is filled with a saline-solution phantom [7, 20, 22]. The noise factor (*NF*), is a simple way to understand the implication of this reduction. If the sample noise dominates then *NF* can be defined as [30]:

9 $$\begin{aligned} \displaystyle NF=20\log _{10}\left[ \frac{\mu (\textrm{B}_{1})-\sigma (\textrm{B}_{1})}{\sqrt{\frac{1}{n}\sum _{s}\mathrm {B_{1}^2}(x,y)}}\right] \end{aligned}$$

where $\mu (\textrm{B}_{1})$ is the mean and $\mathrm {\sigma }(\textrm{B}_{1})$ standard deviation, *n* is the number of image voxels, and *s* is the image space.

## Results and discussion

**Fig. 3:**
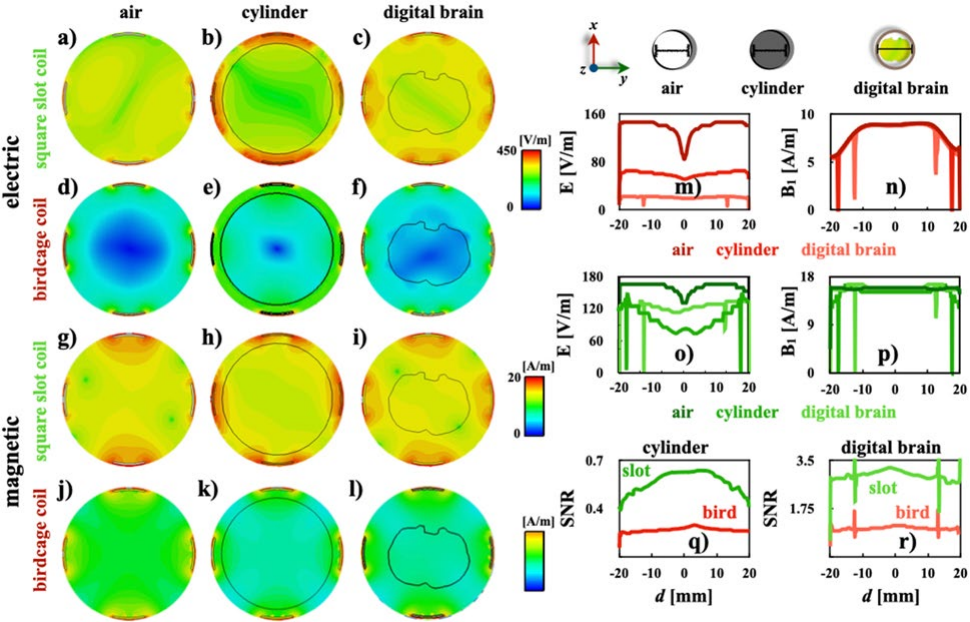
Series of two-dimensional maps of the electric (**a**–**f**) and magnetic (**g**–**l**) fields for the slotted end-ring and birdcage coils, respectively. Comparison plots for E: birdcage coil (**m**) and slotted end-ring coils (**o**), ${\hbox {B}}_1$: birdcage coil (**n**) and slotted end-ring coils (**p**), and SNR for the saline solution (**q**) and the rat’s brain (**r**) phantoms.Plots were computed with data obtained along the black lines across the circles at the top column

Our coil design was validated by full wave electromagnetic simulations, theoretical and experimental sensitivity and phantom images. To examine our coil design in more detail, electromagnetic field simulations included the electric and magnetic field intensity maps of birdcage and slotted-end ring coils for the following loading cases: a) air-filled, b) saline solution-filled phantom and c) rat’s digital brain phantom. Figure 3 shows the two-dimensional maps of the electric (E) and magnetic field (B_1_) for the three cases above, and comparison plots the electromagnetic fields and SNR. All electromagnetic field maps were taken at the mid-section of the RF coils.

 cm

The two-dimensional maps of the magnetic fields of both volume coils have similarities, as shown in Fig. 3g–l. The simulated electromagnetic field results of both coil designs for the saline solution phantom corroborate to: (a) those results reported by Webb [31], (b) numerical evaluations of a band-pass-birdcage coil (ratio = 1 and 8 rungs) computed with a finite element modeling at 127.74 MHz [32], (c) a quadrature low-pass birdcage coil (ratio = 0.7 and 32 rungs) obtained at 7 T for small animal MRI [33] (coil dimensions are essentially the same as ours in this study), (d) numerical results obtained using the finite-difference time-domain method for a low-pass birdcage coil (ratio = 0.7 and 8 rungs) driven in quadrature mode at 7 T for rodents [34], (e) a quadrature birdcage coil (ratio = 0.88 and 16 rungs) for MRI of rabbits at 7 T [35], and (f) various birdcage coils (ratio = 0.62 and 8 rungs) with different rung cross sections at 9.4 T [36]. The B_1_ pattern of the saline solution simulations for both coil designs (Fig. 3n) shows good concordance with simulations obtained at 200 MHz and 400 MHz using a simple two-dimensional full wave model developed by Spence and Wright [37], and similar values of B_1_ and field pattern were reported by Doty et al. for a 2.5 cm Litzcage at 300 MHz [37]. The two-dimensional representations of the electric field for both coil designs in Fig. 3a–f are able to produce the expected behaviour as reported in [20, 32]. The patterns produced by both coils are quite comparable regardless of whether the coil is filled with a phantom or not. However, the air-filled coil case shows the best agreement. The pattern of the electric field reported by Kangarulu et al. [39] for a transverse electromagnetic (TEM) coil with resonant frequency of 340 MHz confirms the patterns of the electric field of both coil designs in Fig. 3m and n. Additionally, results of Fig. 3n and p corroborate the theoretical sensitivity results obtained with eq. (8). Profiles of Fig. 3m and o show that the electric field of the birdcage coil has a greater intensity when compared to our coil design. It is important to mention that a larger amount of electrical energy was absorbed by the birdcage coil when the coil is empty as shown in Fig. 3m. However, Fig. 3m and o show that similar energy levels are absorbed for the rat’s brain and cylinder phantoms. The simulated SNR was computed using the two-dimensional maps of the $\textrm{B}_{1}$ and E. Comparison plots were computed and shown in Fig. 3q and r. These SNR profiles depict a clear numerical improvement of the slotted end-ring coil over the standard birdcage coil for both cases. Following the simulated data of the electromagnetic fields and the methodology proposed in [40], we calculated plots of the electric field as a function of the $\textrm{B}_{1}$ field.

**Fig. 4:**
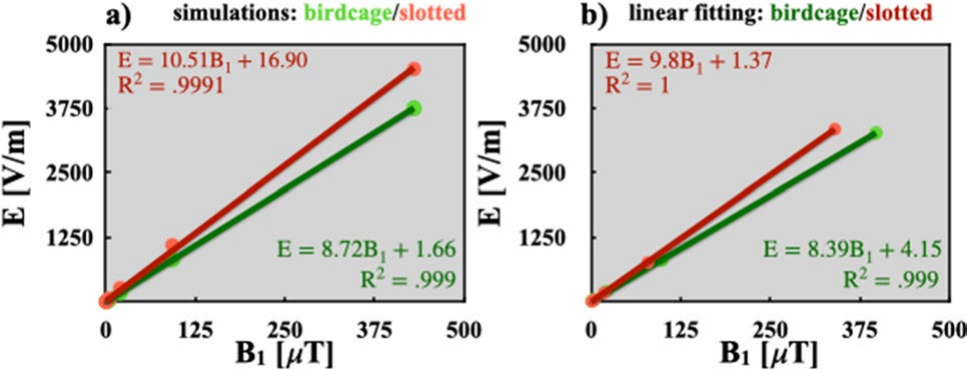
The linear relation between the E and ${\hbox {B}}_1$ magnitudes of the birdcage coil and the slotted end-ring resonator for: **a** saline solution phantom and **b** digital brain phantom

Figure 4 illustrates the plots for the saline solution and digital brain phantoms. As expected, from this we can observe that there is a linear relation between the E and $\textrm{B}_{1}$, and the slope magnitudes are very similar to the experimental results obtained using an electro-optic probe at 128 MHz and 200 MHz. Another important fact is that regardless of the type of phantom used the slope value is practically the same. These experimental results validate the simulations of the electric field produced by the slotted end-ring resonator.

**Fig. 5:**
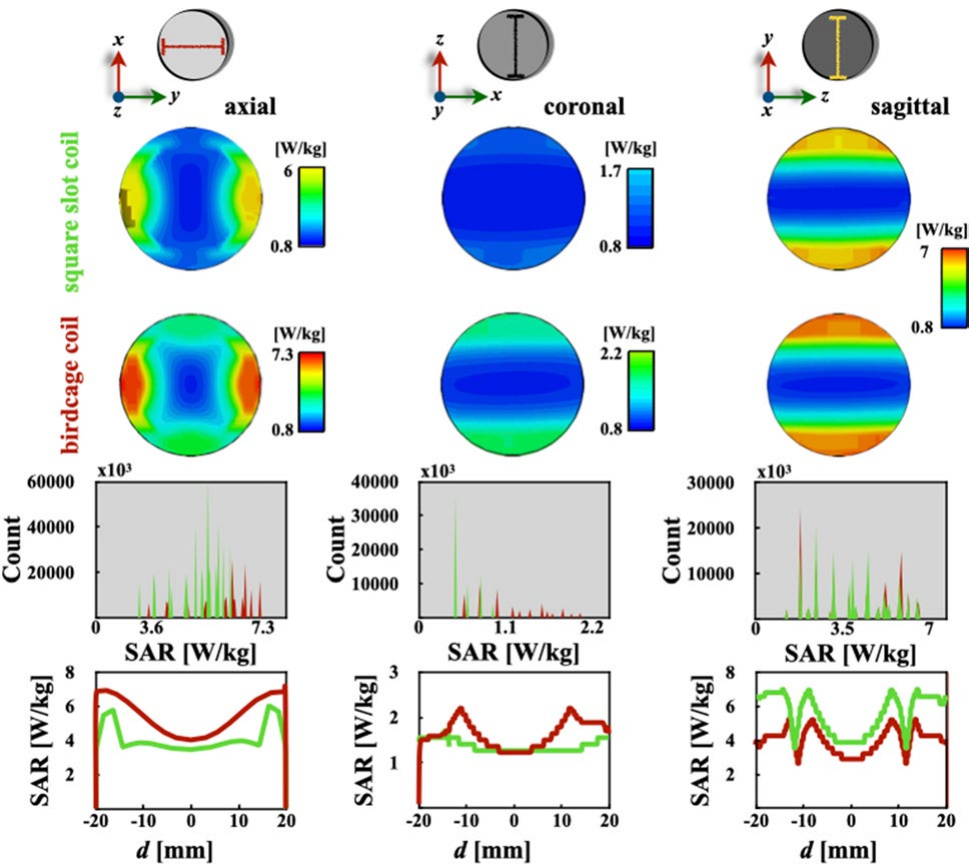
Series of two-dimensional maps of $\mathrm {SAR_{1g}}$ for the two volume coils (**a**–**f**). Simulation data were used to compute for comparison histograms (**g**–**i**) and $\mathrm {SAR_{1g}}$ plots (**j**–**l**) as indicated in the top row illustrations

Numerical modeling of the interaction between the electromagnetic fields and the animal model provides a useful way to assess the rate of energy deposition. The $\mathrm {SAR_{1g}}$ (1 g averaging SAR) numerical assessments for the saline solution and digital brain phantoms are shown in Fig. 5. Additionally, to compare the $\mathrm {SAR_{1g}}$ results, comparison histograms and profiles were obtained for the both phantoms and the standard orientations. The $\mathrm {SAR_{1g}}$ two-dimensional maps of Fig. 5a–f show a very good concordance with two-dimensional maps of SAR obtained via $\textrm{B}_{1}$ mapping of a quadrature birdcage coil (diameter 60 cm) loaded with solution-filled cylinders at 64 MHz T [40]. These $\mathrm {SAR_{1g}}$ predictions in the axial (Fig. 5a and d) and the coronal (Fig. 5b and e) orientations agree very well with results obtained via the tomographic method and a birdcage coil (ratio = 0.91 and 16 rungs) tuned a 128 MHz [42] as well as phantom imaging studies conducted by Cline et al. [43]. 

**Fig. 6:**
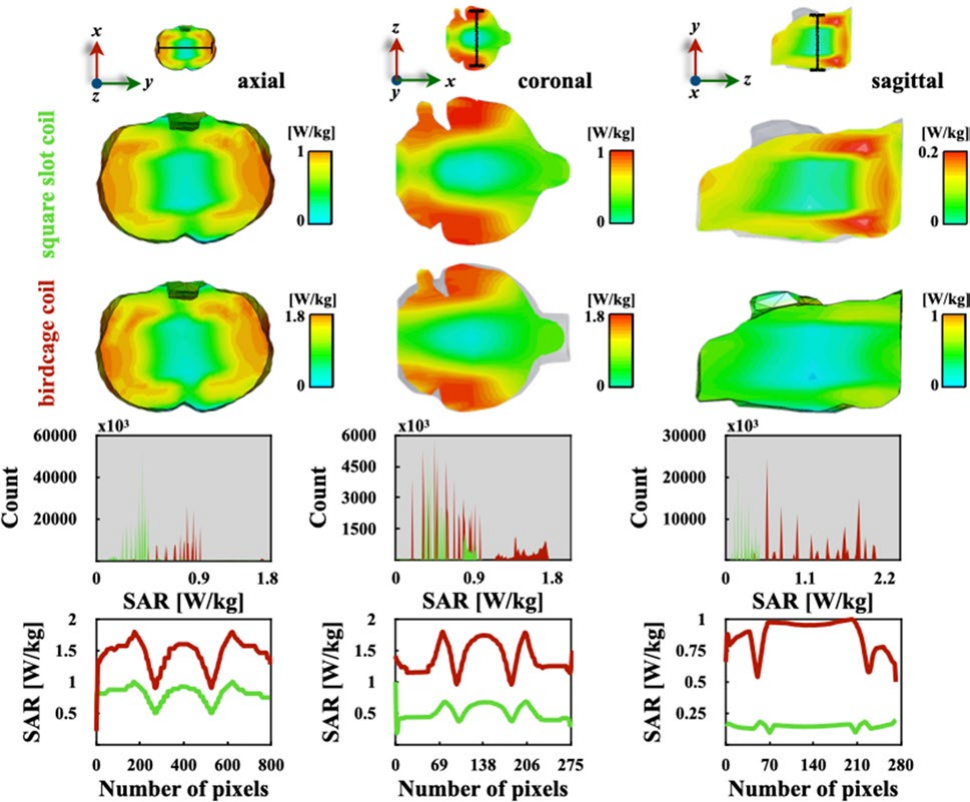
Series of two-dimensional maps of $\mathrm {SAR_{1g}}$ in different orientations for the two volume coils (**a**–**f**). Simulation data were used to compute for comparison histograms (**g**–**i**) and $\mathrm {SAR_{1g}}$ plots (**j**–**l**)

There is a reasonable agreement between the theoretical results published by Hoult for a quadrature-driven volume coil at 200 MHz [44], and the comparison plot for axial orientation of Fig. 5j. Histograms in Fig. 5g–i show that the birdcage coil has a distribution with higher values of $\mathrm {SAR_{1g}}$ compared to our coil design for all three directions. The coronal cut histogram of Fig. 5g has a wider distribution of significantly higher values for the birdcage coil, and the slotted end-ring coil has a much lower rate of absorbed energy for the same interval. Figure 5h shows that the absorbed energy ratio by the slotted volume coil is less than 1.1 W/kg, and the birdcage coil shows higher values for a wider interval approximately between 0.55 and 2 W/kg. The Fig. 4i distribution of energy absorbed rate by the phantom looks roughly the same along the same interval, which is confirmed by the comparison plot in Fig. 5l. Comparison plots of $\mathrm {SAR_{1g}}$ in Fig. 5j–l show practically the same pattern and intensity for both coil designs. However, the birdcage coil shows a slight increase over the slotted end-ring coil in the axial and sagittal orientations; see Fig. 5j and l. This is more easily appreciated at both ends, while towards the centre, the $\mathrm {SAR_{1g}}$ intensity tends to be roughly the same. Similarly, numerical assessments of $\mathrm {SAR_{1g}}$ for the rat’s digital brain phantom were computed to obtain more realistic results. Figure 6 shows results of $\mathrm {SAR_{1g}}$ for three different orientations. These two-dimensional maps correspond very well with simulated (100 mg averaging) $\mathrm {SAR_{100mg}}$ predictions reported by Martin et al. [18]. The $\mathrm {SAR_{1g}}$ intensity values agree with results reported by Wang et al. [44], which assumes electromagnetic plane waves and uses the (Finite-Difference Time-Domain) FDTD method.

The $\mathrm {SAR_{1g}}$ and absorbed power results obtained from simulations in Fig. 6 are summarised in Table 1.

**Table 1:** Comparison of $\mathrm {SAR_{1g}}$ results and the locations of max$\mathrm {SAR_{1g}}$

	Birdcage coil	Slotted-end ring coil
Total ${\scriptstyle {\scriptscriptstyle \mathrm {SAR_{1g}}}}$ [W/kg]	1.20	0.46
Max${\scriptstyle \mathrm {{\scriptscriptstyle SAR_{1g}}}}$ [W/kg]	1.79	0.51
Max${\scriptscriptstyle \mathrm {SAR_{1g}}}$/total ${\scriptscriptstyle \mathrm {SAR_{1g}}}$	1.49	1.10
Max$\mathrm {{\scriptscriptstyle {\scriptstyle SAR_{1g}}}}$ location (*x,y,z*) [mm]	(0.46, –16.51, 47.33)	(–3.25, –8.37, 15.61)

**Fig. 7:**
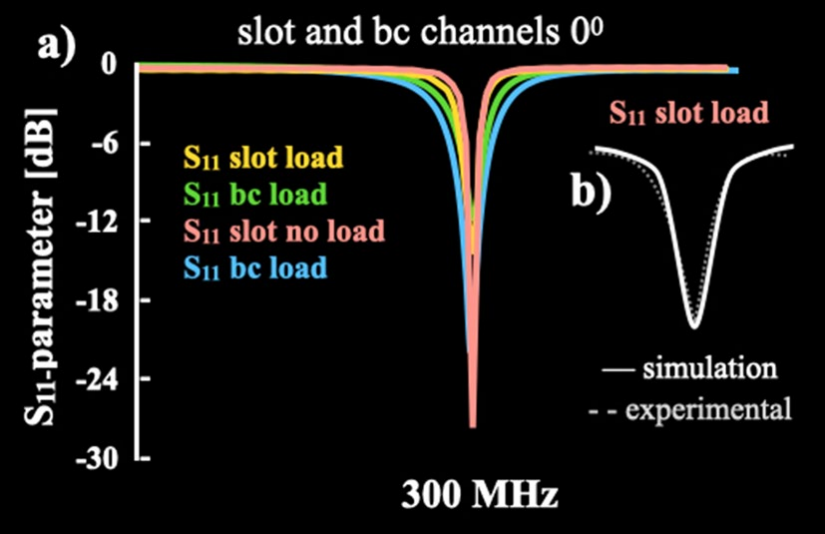
**a** S-parameters of the slotted volume coil comparison before and after loading for the simulation and experimental case. **b** Comparison plots of theory-versus-experiment $\mathrm {S_{11}}$-parameter

$\mathrm {SAR_{1g}}$ predictions of the rat’s brain phantom for the slotted volume coil are lower than 0.5 W/kg in the axial and sagittal orientations as shown in histograms of Fig. 6g and i. In the coronal cut of Fig. 6b, the $\mathrm {SAR_{1g}}$ values are lower than 0.9 W/kg, and the birdcage coil produces a higher ratio for the entire interval. Comparison plots of Fig. 6j–l clearly show lower $\mathrm {SAR_{1g}}$ values of the slotted volume coil than the ones obtained with the birdcage coil for all three cuts. However, the highest difference is in the saggital direction as illustrated in Fig. 6l. The simulated absorbed power values of both coil are in reasonable concordance with the theoretical values obtained with the analytical model of birdcage resonators at similar resonant frequencies and reported by Foo et al. [46]. The $\mathrm {SAR_{1g}}$ two-dimensional mappings of Fig. 6a–f) and their corresponding histograms (Fig. 6g–i) confirm the simulated results obtained with a digital anatomical model of the Sprague-Dawley rat (voxel dimension $1.95\,\textrm{x}\,1.95\,\textbf{x}\,2.15\,\textrm{mm}^{3}$), two-dimensional based on MRI data and the FDTD numerical approach [47]. These calculations show that the slotted volume coil has better agreement with this analytical model despite our brain model having a much lower resolution ($0.18\,\times \,0.18\,\times \,0.5\,\textrm{mm}^{3}$) [18]. Similar power absorption can be observed for the saline solution phantom when compared to the digital brain phantom for both coils. As expected, the use of digital phantoms of specific organs provide more realistic results, despite the fact that our BREP brain phantom is not able to reproduce accurately complex anatomical details. The numerical assessments of the saline solution phantom $\mathrm {SAR_{1g}}$ are practically the same for both coil designs. This is expected because both coil designs have similar topologies. However, the slotted volume coil produces a much lower $\mathrm {SAR_{1g}}$ value when using the digital brain phantom. Something very similar happens for the max$\mathrm {SAR_{1g}}$ calculations which are also in good agreement with values obtained using a probabilistic approach [48]. From the max$\mathrm {SAR_{1g}}$ results, we can observe that both coils have roughly the same values for the saline solution phantom, and when the digital brain model was used as a load, a 3.5-fold increase was produced. These max$\mathrm {SAR_{1g}}$ computations corroborate very well with those computed by Trakic et al. [49] using a Sprague Dawley rat model and a birdcage resonator (ratio = 1) operating at 500 MHz. The location of max$\mathrm {SAR_{1g}}$ values are in the same quadrant with exception of the case for the slotted volume coil and the digital brain phantom. The noise figures of both coil prototypes were computed according to eq. (9), giving $NF\mathrm {_{slot}}\approx 1$, and $NF\mathrm {_{bird}}\approx 1.4$. These two noise figure values have an adequate concordance with those theoretical results for half birdcage and U-shaped split birdcage resonators reported by Gasson et al. [30]. This a reasonable reduction of *NF*: $NF\mathrm {_{bird}}\approx 1.2NF\mathrm {_{slot}}$, is due to B$_{1}$ improvement as shown in Eq. (8). To characterise our coil design, the S-parameters and the Smith chart were experimentally measured. The S-parameter plots and Smith charts of both channels are in Fig. 7. Additionally, S$_{11}$, S$_{12}$ and S$_{21}$ parameters were numerically and experimentally computed for the slotted volume coil of Fig. 1c. This S-parameter comparison has a major concordance between numerical simulations and experimental bench testings. Thus, reliable conditions can be obtained to guide the design of new RF resonators [18, 50]. 

Both channels showed a good RF penetration and impedance values. Impedance values are in good agreement with values reported by [51] and provides an optimal energy transfer. These parameters show a value lower than –20 dB, confirming a good 50-$\Omega$ match and low decoupling between the two channels to drive the coil in quadrature mode [31]. So, these S_11_-parameters profiles show a good isolation of both channels to assure optimal energy transmission and reception of the RF signals. These results are in very good agreement with those reported in [22]. All the bench testing results are summarised in Table 2.

**Table 2:** Bench testing values for both channels of our volume coil prototype

Channel	*Q* factor	RF penetration [dB]	Impedance [$\Omega$]
loaded/unloaded			
$0^{0}$	96/125	$-$35.84/$-$44.63	51.42/49.69
$90^{0}$	81/119	$-$38.63/$-$40.95	51.30/49.83

**Fig. 8:**
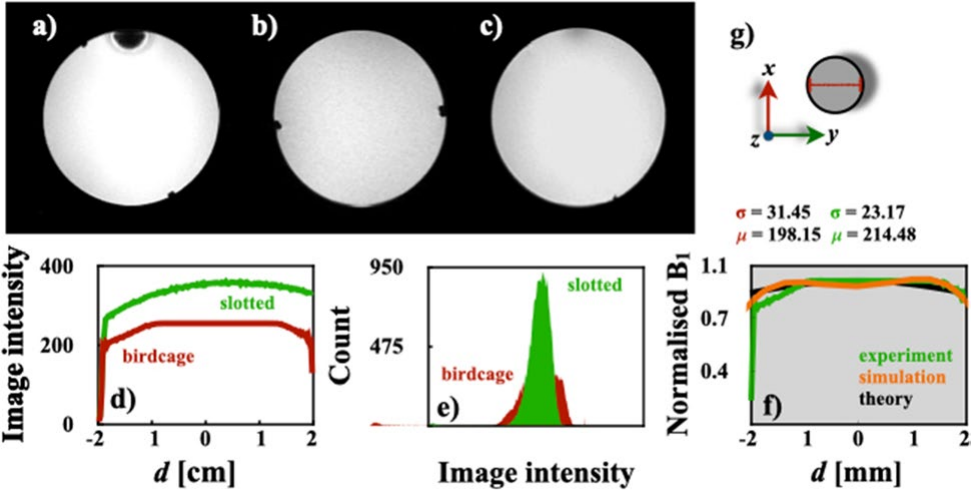
Phantom images: **a** birdcage coil (in-plane resolution = 317.54 $\mu$m x 317.4 $\mu$m x 1 $\mathrm {mm^{3}}$) and, **b** slotted end-ring resonator (in-plane resolution = 312.5 $\mu$m x 312.5 $\mu$m x 1 $\mathrm {mm^{3}}$), **c** B_1_ mapping of slotted coil, **d** comparison uniformity profile of experimental data for the birdcage coil prototype of Fig. 1d and the slotted end-ring coil, **e** Phantom images histograms (**a**, **b** and **f**) comparison of uniformity plot for simulation, theoretical and experimental results. All profiles were taken along the red line in **g**

These *Q* values are in very good concordance with those reported by Marrufo et al. [18]. The slotted end-ring coil shows a slightly better performance than a similar coil previously published with larger dimensions [17]. Phantom images were also acquired for both volume coil designs: Fig. 8a and b shows a comparison of axial images of the spherical phantom acquired with a birdcage coil and our coil, respectively. Comparison of uniformity and histograms for both constructed coils were also calculated using the image data of Fig. 8a and b and shown in Fig. 8d and e. The histogram of the slotted end-ring coil clearly shows a better performance over the traditional birdcage coil. It is important to compare the simulated and theoretical results to experimentally measured B_1_ mapping [31]. We computed the corresponding B_1_ mapping of the slotted coil [20–22], see Fig. 8c. Profiles of B_1_ magnitude were computed using the simulation, theoretical (Eq. (8)) and experimental B_1_ mapping, and a comparison plot was computed, see Fig. 8f. All profiles produced an important concordance to experimentally validate the simulation and theoretical results. The profile patterns also show an excellent agreement with those reported for a birdcage coil tuned and matched to 128 MHz [52].

**Fig. 9:**
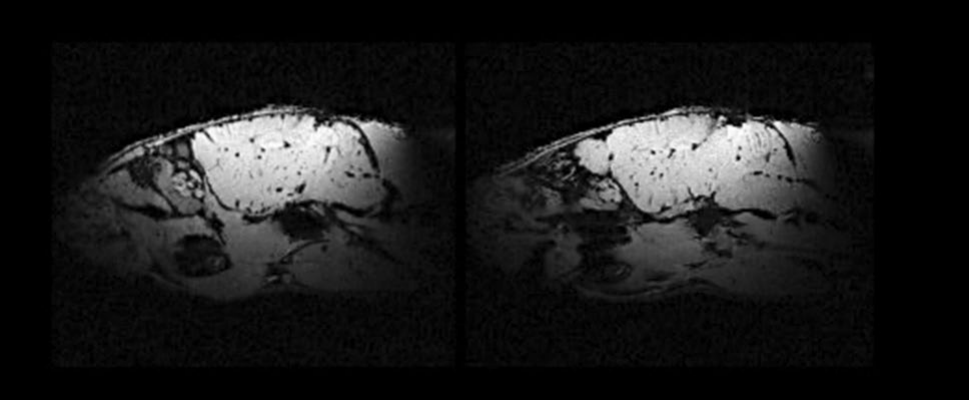
In vivo measurements (in-plane resolution = 117.2 μm × 117.2 μm × 1 $\mathrm {mm^{3}}$) using the slotted end-ring coil and standard spin echo sequences. The anatomical structures of the rat’s brain can be clearly identified

The SNRs were also calculated using the image data of Fig. 8a and b [53]. The SNR values for the coils were approximately 25.43 (slotted end-rings coil) and 17.6 (birdcage coil). The coil design proposed here is able to produce a reasonable improvement on performance over a standard birdcage coil. Consequently, the phantom image acquired with our coil prototype shows a better quality image and good uniformity compared to the image obtained with the birdcage coil. Successful ex vivo results of a rat’s brain were obtained with our volume coil prototype at 300 MHz and shown in Fig. 9. These images of the Wistar rat head show specific brain structures with a high signal intensity, excellent image uniformity, and no movement artifacts. The phantom and rat’s head images prove the compatibility of the slotted end-ring resonator with standard pulse sequences at 300 MHz.

## Conclusion

An RF volume coil with a slotted end-ring was presented. The important parameters of this coil prototype have been calculated analytically and numerically, and confirmed experimentally on the bench and with the MRI. The slotted end-ring coil demonstrates better coil performance than the birdcage coil in terms of sensitivity, homogeneity, SNR and SAR reduction. We have demonstrated that using full-wave electromagnetic simulations and experiments in the Varian 7 T MR imager, the slotted end-ring coil can outperform the birdcage resonator for MRI of small rodents. This can prove to be advantageous when performing functional MRI of rats’ brain where the coil performance plays an important role in acquiring optimal MR signals.
